# Investigating Discontinuity of Age Relations in Cognitive Functioning, General Health Status, Activity Participation, and Life Satisfaction between Young-Old and Old-Old Age

**DOI:** 10.3390/ijerph13111092

**Published:** 2016-11-05

**Authors:** Andreas Ihle, Daniela S. Jopp, Michel Oris, Delphine Fagot, Matthias Kliegel

**Affiliations:** 1 Department of Psychology, University of Geneva, Boulevard du Pont d’Arve 40, Geneva CH-1211, Switzerland; Matthias.Kliegel@unige.ch; 2 Center for the Interdisciplinary Study of Gerontology and Vulnerability, University of Geneva, route des Acacias 54, Carouge CH-1227, Switzerland; Michel.Oris@unige.ch (M.O.); Delphine.Fagot@unige.ch (D.F.); 3 Department of Psychology, University of Lausanne, Geopolis Buildin, Lausanne CH-1015, Switzerland; Daniela.Jopp@unil.ch

**Keywords:** young-old adults versus old-old adults, cognition, health, activity participation, life satisfaction

## Abstract

Health research suggests that findings on young-old adults cannot be generalized to old-old adults and thus that old-old age seems not a simple continuation of young-old age due to qualitative changes that result in a discontinuity in old age. Specifically, it would be of conceptual and methodological importance to inform research regarding estimates around which chronological age the beginning of old-old age could be placed at a population level, and whether this is universal or domain-specific. To derive such criteria, we investigated potential discontinuity of age relations between young-old and old-old age in a large population-based sample considering measures in different domains (processing speed, verbal abilities, general health status, activity participation, and life satisfaction). For processing speed, verbal abilities, general health status, and life satisfaction we observed some very small indication that there might be a discontinuity of age relations at the end of individuals’ eighties, and for activity participation already at the beginning of individuals’ eighties. In conclusion, models conceptualizing aging as a gradual development might not suffice to adequately represent the differences between the stages of young-old and old-old age due to some very small indication that there might be discontinuity in late adulthood.

## 1. Introduction

A key question in research on many health domains in old age concerns whether there is continuity across old age or whether we need to differentiate between a young-old and an old-old age due to discontinuity [1]. The rationale underlying such discontinuity between young-old and old-old age is based on the conceptual view that in young-old age, individuals are in relatively good health and thereby still have, on average, rather substantial amounts of resources that help to compensate age-related losses. Yet, in old-old age individuals’ health resources become fewer and may reach a critical level that no longer allows for compensating age-related losses [1,2]. For example, such losses in health resources by increases in physical health constraints and an increasing number of chronic diseases have been found to affect several aspects of aging such as activity participation and life satisfaction [3]. Evidence for the conceptual view that resource loss can lead to discontinuity in old age comes from studies suggesting that findings on young-old adults cannot be generalized to old-old adults. Thus, old-old age is not a simple continuation of young-old age. For example, in contrast to young-old age there are losses in cognitive functioning such as plasticity, some aspects of learning, and memory in old-old age [4,5,6,7]. While prevalence rates of Alzheimer’s disease are rather low in young-old age (about 2%–3%), they are substantially increased in old-old age (about 40%–50% [8,9]). Overall, functioning seems to be to a degree lower in old-old compared to young-old age, for which a simple linear progression across the entire old-age lifespan would not be sufficient to explain [10,11,12].

To identify the mark that distinguishes between young-old and old-old age, a demographic, population-based approach (which may be limited to developed countries only) represents the chronological age at which 50% of the birth cohort are no longer alive. The logic behind this approach is that people beyond that cutoff age may show substantial losses in multiple health domains [2]. This method would place the beginning of old-old age in developed countries at about 75–80 years [13,14,15]. A major shortcoming of such demographic approaches is that they do not consider measures in specific domains that are important in aging, such as cognitive functioning and health status. Yet, criteria that consider such measures may be of particular importance when the study aim is to compare young-old and old-old adults regarding a certain domain. Lacking such criteria, it is difficult to stratify age groups according to reference categories such as “young-old” and “old-old” age. For a sample selection, researchers have often to return to “practical” strategies such as splitting the available old age sample into two halves. Therefore, in most studies comparing young-old and old-old adults, the decision had to remain rather arbitrary and the old-old age group was stratified to be at least 75 years [7,16,17,18,19], 80 years [10,20,21,22,23,24], or 81 years [25,26], with the young-old age group being respectively below this age. Yet, in comparison to that, in some studies the old-old age group was relatively young, stratified to be at least 70 years [27] or 71 years [28]. In other studies, the old-old age group was even stratified to be more than 85 years [29]. However, for researchers who aim to investigate cross-sectional performance differences between young-old and old-old age, this large variety in sample stratification criteria may cause some problems. For example, for sample selection criteria, it would be necessary to be acquainted with estimations of possible age boundaries to be able to draw valid conclusions regarding the planned comparison of young-old versus old-old adults. But also from a more fundamental perspective, it seems to be important to empirically approach the likely age ranges for those possible qualitative shifts, even or especially when doing so by disentangling different age periods in different domains. So far, solid empirical evidence on those age periods is missing.

Therefore, the present study set out to empirically investigate in detail the pattern of age relations in different domains in a large population-based sample of older adults with a broad age range (65–101 years). Specifically, we investigated the domains cognitive abilities (i.e., processing speed and verbal abilities as markers for fluid and crystallized intelligence), general health status, activity participation, and life satisfaction as they concern important aspects of everyday functioning in old age [1,2,3,4,5,6,7,8,9,10,11,12,22,24,25,26,27,28,29,30,31,32,33,34,35]. For processing speed, verbal abilities, general health status, and activity participation, evidence typically shows a decline in old age, with sharp gradients in old-old age [1,2,3,10,30,31,32]. For life satisfaction, evidence is mixed. While some authors observed an increase with advancing age [3,33], others found a decline in life satisfaction at the end of life [36,37]. To take into account that performance may decline with a larger gradient in a—to be defined—stage of old-old age, commonly a quadratic age term (for the continuous variable “age”) is added to the linear model to represent nonlinear (i.e., accelerated) age relations [30]. Yet, such models still conceptualize aging as a gradual development. Yet, it is questionable whether such gradual (i.e., continuous) functions can adequately represent the hypothesized qualitative differences between the stages of a young-old and an old-old age due to discontinuity in late adulthood [1]. Therefore, our aim was to answer the questions (1) whether there are gradual age relations in the investigated domains across the full range of old age or whether there is a discontinuity of age relations between young-old and old-old age; (2) if so, around which approximate age such discontinuity can be observed at a population level; and (3) whether such discontinuity is universal or domain-specific.

## 2. Materials and Methods

### 2.1. Participants

Data are part of the Vivre-Leben-Vivere (VLV) survey, an ongoing project on vulnerability in Switzerland (LIVES), and were collected in 2011 and 2012. The main (cross-sectional) sample of 3080 participants was randomly selected as a representative sample of the population in the cantonal Swiss administrations’ records and stratified by age (65–69 years, 70–74, 75–79, 80–84, 85–89, and 90+), sex, and canton (i.e., federated states of Switzerland: Basel, Bern, Geneva, Ticino, and Valais). Mean age was 78.4 years (*SD* = 8.4, range 65–101). Further sample characteristics in terms of proportions regarding age groups, sex, and canton are displayed in Table 1. All participants gave their written informed consent for inclusion before they participated in the study. The present study was conducted in accordance with the Declaration of Helsinki, and the protocol had been approved by the ethics commission of the Faculty of Psychology and Social Sciences of the University of Geneva (project identification code: CE_FPSE_14.10.2010).

### 2.2. Materials

#### 2.2.1. Cognitive Abilities

Processing speed. As a marker for fluid intelligence, we assessed the Trail Making Test part A [38], measuring processing speed (i.e., the speed of cognitive processing) and manual dexterity. After seven exercise trails (i.e., connecting the numbers from 1 to 8 with seven lines in total, i.e., 1–2–3–4–5–6–7–8), participants had to connect the numbers from 1 to 25 as fast as possible and without error in ascending order. The latency score was the time in seconds needed to connect the 25 numbers.

Verbal abilities. As a marker for crystallized intelligence, we administered the Mill Hill vocabulary scale [39] measuring verbal abilities (i.e., vocabulary). For each item, participants had to underline the word (which was intermixed with five distractor words) that semantically matched the target word. After one practice item, participants had to complete ten items. The verbal abilities score was the proportion of correctly completed items.

#### 2.2.2. General Health Status

Participants were asked to rate their current general health status based on a scale ranging from 0 = “worst imaginable health” to 100 = “best imaginable health”.

#### 2.2.3. Activity Participation

Participants were asked whether they currently carried out the following 18 leisure activities: (1) go for a walk; (2) gardening; (3) gymnastics or other physical exercises; (4) other sports; (5) go to a café, restaurant, etc.; (6) go to the cinema, theater, etc.; (7) excursions of 1 or 2 days; (8) journeys of at least 3 days; (9) play a musical instrument; (10) other artistic activities; (11) take courses, go to conferences, etc.; (12) party games (cards, scrabble, etc.); (13) crossword puzzles, sudoku, etc.; (14) needlework (knit, dressmaking, etc.); (15) handicrafts, repair, carpentry, pottery, etc.; (16) participation in political or labor union activities; (17) participation in municipality or district activities; and (18) participation in sporting events (e.g., visit a football match, etc.). These activities had been a priori selected with respect to different domains such as mental activities, physical activities, or social activities comprising a large variety of leisure activities [34]. Those activities that were carried out were summed up to derive the overall number of activities individuals engaged in.

#### 2.2.4. Life Satisfaction

We administered the Satisfaction with Life Scale [40]. Participants rated the following five statements “In most ways my life is close to my ideal”; “The conditions of my life are excellent”; “I am satisfied with my life”; “So far I have gotten the important things I want in life”; and “If I could live my life over, I would change almost nothing” using a seven-point Likert scale ranging from −3 (“strongly disagree”) to +3 (“strongly agree”). For analyses, the five item scores were averaged.

### 2.3. Procedure

A face-to-face questionnaire was administered using the CAPI (Computer Assisted Personal Interview) method. This session contained (besides a larger set of other questionnaires) a socio-demographic survey, the items regarding health, activities, and life satisfaction as well as the paper-pencil assessment of the cognitive abilities. Participants were individually tested. The experimenter always assured that the participant fully understood and followed the instructions.

### 2.4. Statistical Analyses

For each inspected domain cognitive abilities/general health status/activities/life satisfaction we examined age relations, testing both a gradual model as well as a stage model and comparing which model provided a better variance explanation. Specifically, applying a gradual model, we regressed the scores of the respective variable on chronological age, testing for linear and quadratic age terms. Applying a stage model, we examined whether age gradients showed differential patterns in young-old versus old-old age. For this purpose, we regressed cognitive abilities/general health status/activities/life satisfaction on chronological age (testing for a linear age term) plus an age group factor (young-old versus old-old) plus an interaction term of the linear age term and the age group factor. Thereby, we used an iterative approach varying the age separating young-old from old-old adults to identify the stage model that descriptively explained the largest amount of variance. We then tested whether this stage model significantly increased explained variance compared to the gradual model (i.e., by evaluating the difference in the respective model test statistic in relation to the difference in degrees of freedom between the two contrasted models, testing for significance). If so, this would suggest that there may be no pattern of gradual age relations across the full range of old age due to a discontinuity of age relations between the stages of young-old and old-old age. In this stage model, a significant age group factor would indicate that mean scores of young-old versus old-old adults significantly differ (i.e., we centered age groups on chronological age to be able to compare average-score differences between the two stages). A significant interaction of the age group factor with the linear age term would indicate that age relations show differential patterns in young-old versus old-old age (i.e., reflecting age-gradient differences between the two stages). If such interactions were significant, we examined in a follow-up analysis age relations for each age group (young-old/old-old) separately by regressing the respective variable on chronological age (testing for a linear age term) in each age group.

To ensure that higher values represented better performance across all variables (as it is common in correlative studies), for processing speed the distribution of latency scores of all participants was reversed based on the sample mean so that interindividual differences remained identical. We conducted analyses with processing speed based on 2073 participants, analyses with verbal abilities based on 2812 participants, and all other analyses based on 3080 participants. Note that in the extensive survey procedure, we had administered the cognitive tests only if there was enough time left, allowing a proper administration of the verbal abilities test in a total of 2812 participants. Note also that we had administered the processing speed test only if the participant had properly performed all seven exercise trails. Furthermore, we terminated the processing speed test (without any score) when the individual made any error in connecting the 25 numbers, allowing a proper administration of the processing speed test in 2073 participants. We applied these restrictive criteria to be able to directly compare reaction times (i.e., reaction times would be confounded when including participants who made errors and took additional time to correct them). We evaluated whether due to these restrictive criteria there were differences between the remaining sample and those individuals who were not included. Compared to the individuals who were not included, the remaining sample was slightly younger (included: *M* = 78.14 years, *SD* = 8.15; not included: *M* = 78.79 years, *SD* = 9.02, *p* = 0.043), had a better general health status (included: *M* = 76.05, *SD* = 19.31; not included: *M* = 73.54, *SD* = 21.67, *p* = 0.001), carried out a greater number of activities (included: *M* = 8.37, *SD* = 3.41; not included: *M* = 7.70, *SD* = 3.66, *p* < 0.001), but did not differ in terms of life satisfaction (included: *M* = 1.38, *SD* = 1.05; not included: *M* = 1.36, *SD* = 1.13, *p* = 0.720). For all analyses, the R environment [41] was used.

## 3. Results

### 3.1. Descriptive Statistics

Table 2 shows means and standard deviations of scores in cognitive abilities, general health status, activities, and life satisfaction.

### 3.2. Examining the Pattern of Age Relations across Old Age

#### 3.2.1. Processing Speed

**Figure 1:**
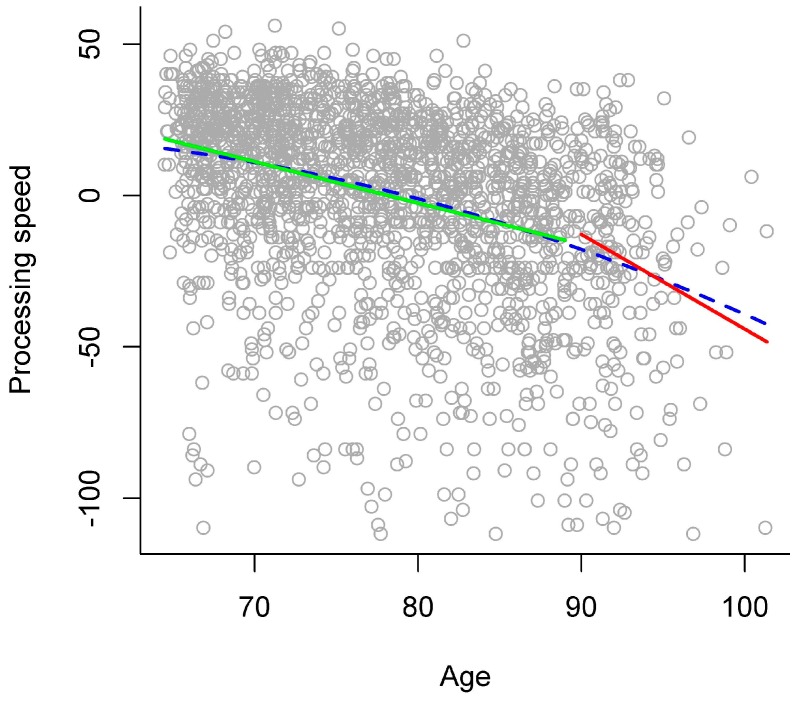
Age relations in processing speed. Scores in processing speed plotted against chronological age, with different models of age relations: gradual model (blue dashed line) and stage model (young-old adults: green solid line, versus old-old adults: red solid line). Note that to achieve that higher values represented better performance across all variables, the distribution of latency scores was reversed. Individuals with negative values were slower than the mean.

In the gradual model, higher chronological age was significantly related to lower processing speed (see left panel of Table 3 and blue dashed line in Figure 1 for an overview). The stage model that descriptively explained the largest amount of variance separated the stages of young-old versus old-old adults at age 90. There was no difference in explained variance between this stage model and the gradual model (see right panel of Table 3). In this stage model, processing speed was significantly lower in old-old than in young-old adults (see right panel of Table 3). Most importantly, to evaluate differences between young-old and old-old adults, there was a significant interaction of age group with the linear age term, indicating that the two age groups differed regarding the relation of processing speed with chronological age. Thus, examining age relations for each age group separately in a follow-up analysis revealed that higher chronological age was significantly related to lower processing speed in young-old adults (*b* = −1.37, *p* < 0.001), but with a much steeper gradient in old-old adults (*b* = −3.13, *p* < 0.001; see green and red solid line in Figure 1).

#### 3.2.2. Verbal Abilities

**Figure 2:**
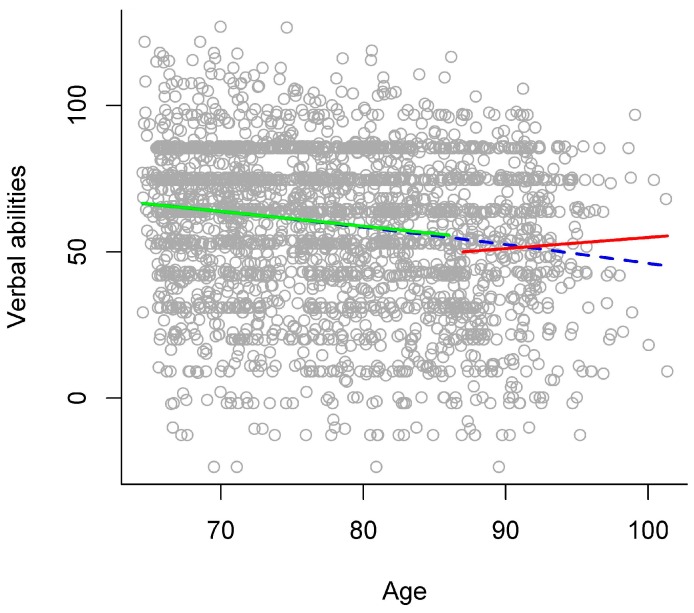
Age relations in verbal abilities. Scores in verbal abilities plotted against chronological age, with different models of age relations: gradual model (blue dashed line) and stage model (young-old adults: green solid line, versus old-old adults: red solid line).

In the gradual model, higher chronological age was significantly related to lower verbal abilities (see left panel of Table 3 and blue dashed line in Figure 2 for an overview). The stage model that descriptively explained the largest amount of variance separated the stages of young-old versus old-old adults at age 87. In comparison to the gradual model, this stage model significantly increased explained variance to a very small extent (see right panel of Table 3), thereby indicating that there might be a discontinuity of age relations around age 87 between young-old and old-old adults. In this stage model, verbal abilities were significantly lower in old-old than in young-old adults (see right panel of Table 3). In addition, there was a significant interaction of age group with the linear age term, indicating that the two age groups differed regarding the relation of verbal abilities with chronological age. Thus, examining age relations for each age group separately in a follow-up analysis revealed that higher chronological age was significantly related to lower verbal abilities in young-old adults (*b* = −0.50, *p* < 0.001), but not in old-old adults (*b* = 0.38, *p* = 0.346; see green and red solid line in Figure 2).

#### 3.2.3. General Health Status

**Figure 3:**
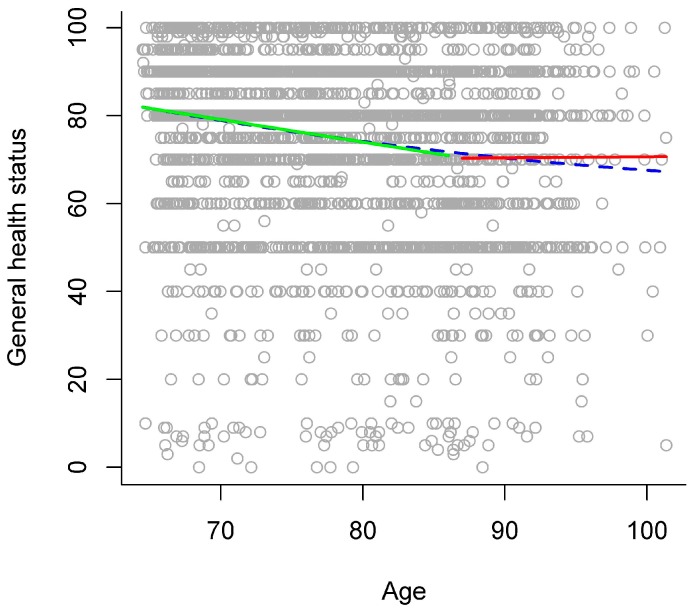
Age relations in general health status. Scores in general health status plotted against chronological age, with different models of age relations: gradual model (blue dashed line) and stage model (young-old adults: green solid line, versus old-old adults: red solid line).

In the gradual model, higher chronological age was significantly related to lower general health status (see left panel of Table 3 and blue dashed line in Figure 3 for an overview). The stage model that descriptively explained the largest amount of variance separated the stages of young-old versus old-old adults at age 87. In comparison to the gradual model, this stage model significantly increased explained variance to a very small extent (see right panel of Table 3), thereby indicating that there might be a discontinuity of age relations around age 87 between young-old and old-old adults. In this stage model, general health status was significantly lower in old-old than in young-old adults (see right panel of Table 3). In addition, there was a significant interaction of age group with the linear age term, indicating that the two age groups differed regarding the relation of general health status with chronological age. Thus, examining age relations for each age group separately in a follow-up analysis revealed that higher chronological age was significantly related to lower general health status in young-old adults (*b* = −0.51, *p* < 0.001), but not in old-old adults (*b* = 0.02, *p* = 0.933; see green and red solid line in Figure 3).

#### 3.2.4. Activity Participation

**Figure 4:**
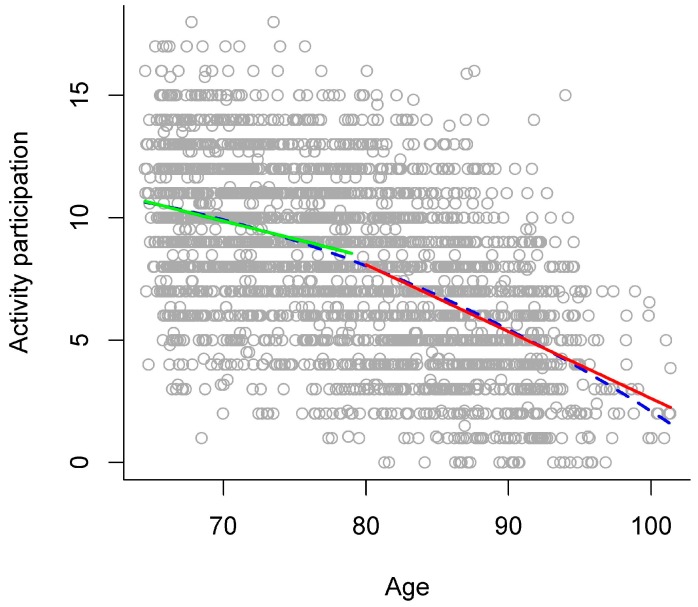
Age relations in activity participation. Number of activities plotted against chronological age, with different models of age relations: gradual model (blue dashed line) and stage model (young-old adults: green solid line versus old-old adults: red solid line).

In the gradual model, higher chronological age was significantly related to a lower number of activities (see left panel of Table 3 and blue dashed line in Figure 4 for an overview). The stage model that descriptively explained the largest amount of variance separated the stages of young-old versus old-old adults at age 80. In comparison to the gradual model, this stage model significantly increased explained variance to a very small extent (see right panel of Table 3), thereby indicating that there might be a discontinuity of age relations around age 80 between young-old and old-old adults. In this stage model, the number of activities was significantly lower in old-old than in young-old adults (see right panel of Table 3). In addition, there was a significant interaction of age group with the linear age term, indicating that the two age groups differed regarding the relation of the number of activities with chronological age. Thus, examining age relations for each age group separately in a follow-up analysis revealed that higher chronological age was significantly related to a lower number of activities in young-old adults (*b* = −0.15, *p* < 0.001), but with a much steeper gradient in old-old adults (*b* = −0.27, *p* < 0.001; see green and red solid line in Figure 4).

#### 3.2.5. Life Satisfaction

**Figure 5:**
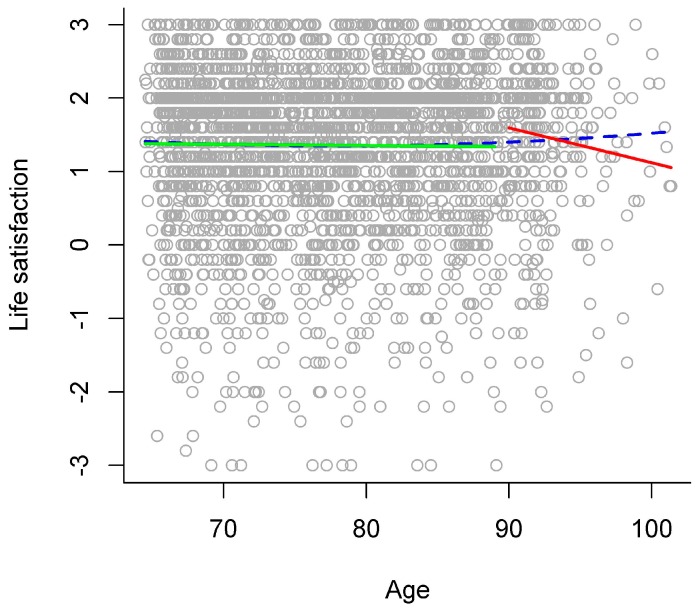
Age relations in life satisfaction. Scores in life satisfaction plotted against chronological age, with different models of age relations: gradual model (blue dashed line) and stage model (young-old adults: green solid line versus old-old adults: red solid line).

In the gradual model, chronological age was not related to life satisfaction (see left panel of Table 3 and blue dashed line in Figure 5 for an overview). The stage model that descriptively explained the largest amount of variance separated the stages of young-old versus old-old adults at age 90. In comparison to the gradual model, this stage model significantly increased explained variance to a very small extent (see right panel of Table 3), thereby indicating that there might be a discontinuity of age relations around age 90 between young-old and old-old adults. In this stage model, there was no difference in life satisfaction between old-old and young-old adults (see right panel of Table 3). In addition, there was no interaction of age group with the linear age term. Exploratively examining age relations for each age group separately in a follow-up analysis revealed that chronological age was not related to life satisfaction in young-old adults (*b* = −0.002, *p* = 0.588), but that higher chronological age was significantly related to lower life satisfaction in old-old adults (*b* = −0.05, *p* = 0.048; see green and red solid line in Figure 5).

## 4. Discussion

The present study set out to investigate the pattern of age relations in different domains in a large population-based sample of older adults covering a broad age range. First of all, for the domains verbal abilities, general health status, activity participation, and life satisfaction we observed a very small increase in explained variance with the stage model compared to the gradual model. This indicates that for these domains there may be no pattern of gradual age relations across the large range of old age considered here, but that there may be some very small indication that there might be a discontinuity of age relations between the stages of young-old and old-old age. Note that the gradual models (that were applied as reference models) also considered quadratic age terms acknowledging accelerated decline in older age. Our very small effects observed might confirm the need to conceptually distinguish between a young-old and an old-old age emphasized in the literature [1,2]. Moreover, this is in line with studies suggesting that compared to young-old age, old-old age is related to particular loss [5,7,9,10,11]. Thus, models conceptualizing aging as a gradual (i.e., continuous) development (as it is commonly applied by regressing the respective variable of interest on continuous age terms) may not adequately represent the *differences* between the stages of young-old and old-old age due to possible discontinuity in late adulthood [1,2].

With respect to the questions around which approximate age such discontinuity can be observed and whether this is universal or domain-specific, present results provided some very small indications. For cognitive abilities (processing speed and verbal abilities), general health status, and life satisfaction, we observed some very small indications that there might be a discontinuity of age relations at the end of individuals’ eighties. In comparison, for activity participation we observed some very small indication that there might be discontinuity already at the beginning of individuals’ eighties. For processing speed and activity participation, higher chronological age was related to lower scores within young-old age. Yet, in old-old age scores were lower (compared to young-old age) and the negative link between chronological age and scores stronger than in the young-old group, indicating that age relations were even more pronounced within old-old age. On a general level, this is in line with findings that processing speed and activity engagement decline in old age [30,42]. For verbal abilities and general health status, higher chronological age was related to lower scores within young-old age. In old-old age scores were lower (compared to young-old age) but did not show an age relation within old-old age. This confirms prior findings regarding the maintenance of verbal abilities and subjective health in old age [43,44]. For life satisfaction, there was no age relation within young-old age, but higher chronological age within the old-old age group was related to lower life satisfaction (see, e.g., [36,37,45] for a discussion on the “paradox” of subjective well-being in old age).

Thus, there may be some very small indication that the different domains might follow specific patterns of age relations across old age. This underlines the need to consider domain-specific development when conceptually distinguishing between young-old versus old-old age. Present findings further provide some very small indication to corroborate the view that performance does not simultaneously decrease in all domains at the same time in old age and that functioning may be maintained in some abilities, even if deficits occur in other domains [46,47].

Baltes and Smith [2] noted that it may be difficult to directly link the concept of young-old versus old-old age to a specific age range as there may be variation regarding age at which old-old age begins. Although this seems undoubtedly reasonable, we feel it would be necessary to gain some more insight on estimations of possible age boundaries, for theoretical and practical reasons. Theoretically, this would be essential in order to further advance general models of development in old age and better understand the differences between these life phases. Practically, such age boundaries would help to create sample selection criteria for studies aiming to investigate cross-sectional performance differences between “young-old” and “old-old” adults and for being able to draw valid conclusions regarding the planned comparison of age groups. Note that our aim was not to identify a specific age at which age groups as a whole are expected to start functioning more poorly. Instead, our goal was to derive estimates around which approximate age, on average, the beginning of old-old age could be placed due to differential patterns of age trends compared to young-old age. Thereby, following our results from a large population-based sample of older adults considering different domains, there may be some very small indication that one might place the beginning of old-old age at the end of individuals’ eighties for cognitive abilities (such as processing speed and verbal abilities), general health status, and life satisfaction. For activity participation, there may be some very small indication that one might place the beginning of old-old age at the beginning of individuals’ eighties. Clearly, recruiting adults being 80 or older for demanding experimental studies is challenging and therefore researchers often return to more practical strategies such as splitting the available old-age sample into two halves. Yet, following present results, the common approach in many studies to consider individuals at age 75 or older being of “old-old age” (respectively young-old adults being 74 years or younger) might lead to an underestimation of effect sizes regarding differences between young-old versus old-old age. For studies separating the two age groups already at age 70, this might be rather an inappropriate approach as such bias might be even more pronounced.

As another methodological note, one may argue that considering a quadratic age term (i.e., regarding the continuous variable “age”) in statistical models to represent nonlinear (i.e., accelerated) age relations already takes into account that performance may decline with a larger (or smaller) gradient in old-old age and that this may suffice to fully describe development in late adulthood. Note that such models still conceptualize aging as a gradual development. There may be an indication that this might not suffice to adequately represent the *qualitative differences* between the stages of young-old and old-old age due to some very small evidence that there might be discontinuity in late adulthood. Specifically, age gradients of functioning in old-old age might be better depicted with a stage model approach than with a gradual curve approach. For example, when applying a gradual curve approach, the age gradients of processing speed and activity participation in old-old age might be underestimated or would resemble those gradients only in very late adulthood (see Figure 1 and Figure 4). The gradients of verbal abilities and general health status in old-old age would possibly be overestimated with a gradual curve approach (see Figure 2 and Figure 3). Thus, the estimations of age gradients with a gradual curve approach might be more moderate, “smoothing” the qualitative differences between the stages of young-old versus old-old age, and thereby might not reflect the specific discontinuity patterns observed with a stage model in detail.

We acknowledge that the present study is limited by its cross-sectional design that does not allow for causal inferences. Present analyses give only information about age differences but do not allow drawing conclusions regarding intraindividual changes over time (see also, e.g., [10] for other cross-sectional studies examining differential patterns between young-old and old-old age). Therefore, we cannot say whether a decline in activity participation in old age may be a result of decline in health and cognitive functioning, which needs to be investigated with longitudinal data in future studies. In addition, we acknowledge that present cross-sectional results may be confounded with cohort effects. Yet, Baltes and Smith [2] argue in their framework that historical cohort or generational improvements in health and cognitive functioning are typically much smaller than the aging effects that are related to discontinuity between young-old and old-old age. Nevertheless, present observations await replication in future longitudinal research. Moreover, we acknowledge that the present study is explorative and future studies are needed to confirm the observed patterns in young-old versus old-old age. Thereby, future longitudinal research might investigate the detailed mechanisms in terms of losses and resource depletion that lead to the conceptualized qualitative differences between young-old and old-old age [1,2]. We also acknowledge that the increase in explained variance by applying the stage model, relative to the gradual model, (although significant) was very small for the investigated domains. This may be due to the possibility that with the present population-level approach age trajectories within individuals may be (at least partly) masked by heterogeneity in the threshold age for discontinuity across individuals that lead to large interindividual differences in cognitive functioning and health commonly observed in old age [2,47]. Thereby, in addition to a population approach that considers average development, it may be fruitful to also study the pattern of gains and losses across individuals’ life courses in future longitudinal research, which may also help to disentangle the large interindividual variance in old age commonly observed. Besides that, we acknowledge the general limitation that the present results on general health status and activity participation are based on subjective measures. Hence, findings await replication with objective measures for these domains. Future research might also include more measures of functional health and physical functioning. In addition, we acknowledge the limitation that, for activity participation in old age, we only had information on current activities. We had no information to say how stable individuals’ activity participation was over the past time. This further underlines the need for future longitudinal research on this issue. Moreover, the present study was performed in Switzerland, a country with a relatively high life expectancy, high average socio-economic status and educational level. Thus, we acknowledge that the generalizability of present results to other study populations with lower life expectancy and lower socio-economic status or educational level may be limited. Future research might consider a comparison of different countries to investigate to what extent patterns of discontinuity differ between such populations.

One may argue that the relatively stable patterns in verbal abilities and general health status across old-old age observed with the stage model may underestimate a potential age-related decrease, particularly in very late adulthood. Yet, note that the observed pattern is in line with evidence suggesting that some individuals may be able to maintain verbal abilities and subjective health in old age although there is an average tendency of loss in the population [43,44]. In this context one may further argue that there may not have been enough cases who suffered severe decline. Yet, such a stability pattern may be (at least partly) driven by those who already suffered severe decline and died subsequently, while others who were less vulnerable to age-related losses were still alive. Thereby, mean performance of the population may not decrease. Again, future longitudinal research might investigate such mechanisms in more detail. Moreover, we acknowledge the limitation that the applied processing speed test also measures manual dexterity. Hence, future research may use a set of other cognitive tests, controlling for physical limitations such as poor manual dexterity and tremor in old age. In addition, we acknowledge that due to the restrictive criteria for the processing speed test and the missing values in the verbal abilities test due to time constraints in the testing procedure (see Statistical Analyses section for further details), there was a certain selection of the remaining sample for these measures. It may be possible that individuals who properly did the processing speed and the verbal abilities test had better cognitive functioning, better general health status, carried out more activities, and were slightly younger than those who were not able to properly follow the entire testing procedure within the time limits. Thus, we acknowledge that the generalizability of present results to the entire living population is limited. Future research might investigate whether the present pattern of results holds also for individuals with impairments, which could give further insights into the qualitative differences between young-old and old-old adults.

## 5. Conclusions

In line with the concept of young-old versus old-old age [1], there may be an indication that models conceptualizing aging as a gradual development might not suffice to adequately represent the qualitative differences between the stages of young-old and old-old age due to some very small evidence that there might be discontinuity in late adulthood. In addition, it may be possible that domains might differ regarding the beginning of old-old age and their specific trajectories in aging.

## Figures and Tables

**Table 1:** Descriptive sample characteristics in terms of proportions regarding canton, sex, and age groups.

Stratification Variables	Overall *N* = 3080	
**Age group**	65–69: 573 (18.6%)	70–74: 609 (19.8%)
	75–79: 567 (18.4%)	80–84: 495 (16.1%)
	85–89: 463 (15.0%)	90+: 373 (12.1%)
**Sex**	Women: 1485 (48.2%)	Men: 1595 (51.8%)
**Canton**	Basel: 636 (20.6%)	Bern: 684 (22.2%)
	Geneva: 578 (18.8%)	Ticino: 606 (19.7%)
	Valais: 576 (18.7%)	

The proportions regarding age groups are only for descriptive purposes regarding stratification criteria. In all analyses with chronological age as predictor, it was treated as a continuous variable.

**Table 2:** Means and standard deviations of analyzed measures.

Variable	*M*	*SD*
Processing speed (seconds)	66.20	30.61
Verbal abilities (percent correct)	59.4	25.7
General health status (rating 0 to 100)	75.24	20.13
Activities (number)	8.15	3.50
Life satisfaction (rating −3 to +3)	1.37	1.07

Means and standard deviations of scores in cognitive abilities, general health status, activities, and life satisfaction.

**Table 3:** Age relations in different domains applying a gradual versus applying a stage model.

Variable	Gradual Model			Stage Model					
	*b_linear_*	*b_quadratic_*	*R^2^*	Age Break	*b_linear_*	*b_agegroup_*	*b_interaction_*	*R^2^*	Δ*R^2^*
Processing speed	2.35 ^ns^	−0.02 *	0.14 ***	90	−3.13 ***	22.83 ***	1.76 *	0.14 ***	< 0.001 ^ns^
Verbal abilities	−0.15 ^ns^	−0.003 ^ns^	0.03 ***	87	0.38 ^ns^	9.99 ***	−0.88 *	0.03 ***	0.003 ** (8.1%)
General health status	−1.22 ^ns^	0.005 ^ns^	0.03 ***	87	0.02 ^ns^	6.07 ***	−0.53 *	0.03 ***	0.001 * (4.0%)
Activities	0.36 **	−0.004 ***	0.29 ***	80	−0.27 ***	3.29 ***	0.12 ***	0.29 ***	0.001 * (0.4%)
Life satisfaction	−0.06 ^ns^	0.0004 ^ns^	0.0006 ^ns^	90	−0.05 *	−0.11 ^ns^	0.05 ^ns^	0.003 ^ns^	0.002 * (312.2%)

Age relations in cognitive abilities, general health status, activities, and life satisfaction. Left panel: Regression findings applying a gradual model, with scores in the respective variable regressed on chronological age (testing for linear and quadratic age terms). *b_linear_* = regression coefficient of the linear age term. *b_quadratic_* = regression coefficient of the quadratic age term. *R^2^* = multiple *R^2^*, resulting total variance that is accounted for by the linear and the quadratic age term. Right panel: Regression findings applying a stage model, with scores in the respective variable regressed on chronological age (testing for a linear age term) plus an age group factor (young-old versus old-old) plus an interaction term of the linear age term and the age group factor. Age break = the age separating young-old from old-old age. *b_linear_* = regression coefficient of the linear age term. *b_agegroup_* = regression coefficient of the age group factor. *b_interaction_* = regression coefficient of the interaction term. *R^2^* = multiple *R^2^*, resulting total variance that is accounted for by the linear age term, the age group factor, and the interaction term. Δ*R^2^* represents the amount of increase in explained variance in the stage model compared to the gradual model (in parentheses, the percentage regarding the increase relative to the initial amount of explained variance by the gradual model is given). Higher values represented better performance across all variables. *** *p* < 0.001; ** *p* < 0.01; * *p* < 0.05; ^ns^ non-significant, *p* > 0.05; two-tailed.
